# Technical challenges and outcomes of stereotactic biopsies in the posterior fossa: Experience with ZD-inomed and leksell vantage frames

**DOI:** 10.1007/s00701-024-06329-y

**Published:** 2024-11-20

**Authors:** Insa Prilop, Stephan B. Sobottka, Clara Buszello, Ilker Y. Eyüpoglu, Witold H. Polanski

**Affiliations:** Department of Neurosurgery, Medizinische Fakultät und Universitätsklinikum Carl Gustav Carus, Technische Universität Dresden, Fetscherstraße 74, 01307 Dresden, Germany

**Keywords:** Stereotactic biopsy, Posterior fossa, Stereotactic frame setup, Trancerebellar approach

## Abstract

**Introduction:**

Stereotactic brain biopsies are essential for obtaining tissue samples from brain lesions, crucial for comprehensive histological analysis and subsequent adjuvant therapies. While most biopsies target supratentorial lesions, those involving the posterior fossa are less frequent but pose significant technical and surgical challenges, necessitating careful patient management.

**Methods:**

We present our experience with stereotactic biopsies of the posterior fossa using the Leksell Vantage frame (Elekta, Stockholm, Sweden) and the ZD Inomed frame (Inomed Medizintechnik GmbH, Emmendingen, Germany). For the ZD frame, we either mounted it upside down or employed a frontal approach, while for the Leksell Vantage frame, we utilized a reverse x-axis orientation. Planning was based on 3-T MRI scans and preoperative MRI merged with stereotactic CT for coordinate generation.

**Results:**

From 2006 to 2023, we performed 25 stereotactic biopsies of the posterior fossa in our department—9 with the ZD Inomed frame and 16 with the Leksell Vantage frame. The cohort included 14 male and 11 female patients, with an average age of 60.6 years (range 36—80 years). The average surgery duration was shorter with the Leksell Vantage frame (32.6 min vs. 44.8 min, p = 0.05). The average length of the planned trajectory was 41.7 mm for the Leksell Vantage frame and 52.2 mm for the ZD Inomed frame. Postoperativ bleeding occurred in two cases—one managed conservatively, the other required surgical intervention. Additionally, two other cases presented new postoperative focal neurological deficit. The overall mortality rate was 34.8% and a 40-day postoperative mortality rate of 13.0%.

**Conclusion:**

Our experience demonstrates that stereotactic biopsies of lesions in the posterior fossa can be effectively managed with different frame systems, though they present a higher degree of complexity. Notably, the Leksell Vantage frame was associated with a significantly shorter surgery duration. This technical note provides valuable insights and detailed technical guidance for neurosurgeons facing similar challenges.

## Introductions

Stereotactic brain biopsies are a routinely performed procedure aimed at obtaining representative tissues samples from brain lesions for diagnostic purposes. This method is crucial for providing comprehensive histological analysis, essential for guiding subsequent adjuvant therapies. While the majority of these biopsies target lesions in the supratentorial region, those involving the posterior fossa are less common and present significant challenges due to vulnerable anatomical structures such as the brainstem, cerebellar peduncle and cerebellum. Various lesions can occur in this region, including neoplastic (primary brain tumors or metastasis), radiation-induced necrosis, inflammatory processes and vascular formations (hematoma, ischemic lesions) [3].

Over the past decades the diagnostic value and low complication rates of stereotactic brain biopsies have improved in parallel with advancements in radiological imaging techniques (CT and MRI) [4, 6]. Stereotactic biopsies of the posterior fossa and brainstem began in 1975, initially based on CT scans [10]. There are two primary approaches for accessing the posterior fossa via stereotactic biopsy: the extraventricular frontal approach and the suboccipital transcerebellar approach. Previous studies have noted varying preferences for each approach, though these differences were not statistically significant [3, 5].

When comparing frame-based and frameless approaches, studies have shown that frameless stereotactic biopsies have limitations in the posterior fossa, particularly regarding concerning the rate of accurate histopathological diagnosis [2, 1]. Given these complexities and the technical challenges inherent in stereotactic procedures, careful consideration in patient management is essential.

In this technical note, we share our experiences with stereotactic biopsies of the posterior fossa using both the ZD-Inomed frame and the Leksell Vantage frame. Rather than emphasizing a comparison between these frames, the focus is on providing detailed technical guidance and insights into managing the chalnneges associated with performing biopsies in the posterior fossa. This aims to offer practical value to neurosurgerons navigating these difficult cases.

## Methods

We performed a retrospective single center study involving all adult patients who underwent a stereotactic biopsy of a lesion located in the posterior fossa between March 2006 and January 2024. The included patients had lesions within the cerebellum or the brainstem that were not reasonable accessible through open surgery.

**Fig. 1:**
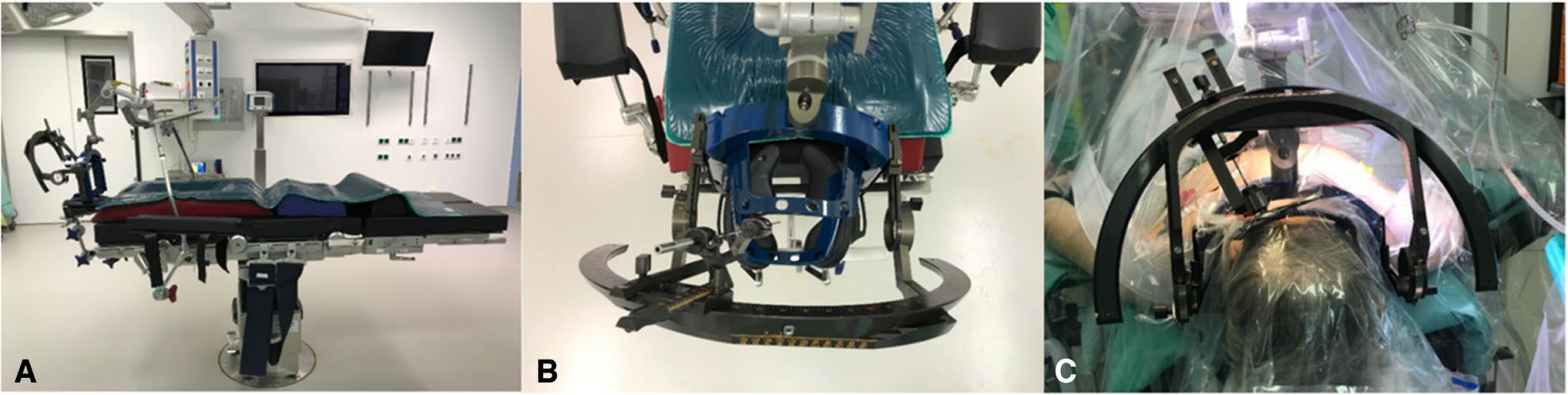
Setting of using the Leksell Vantage frame with dorsal frame attachment to operating table. **A**: Frame setting in the operating theater, **B**: Bird´s eye view of frame setting, **C**: Patient undergoing a biopsy of the posterior fossa

**Fig. 2:**
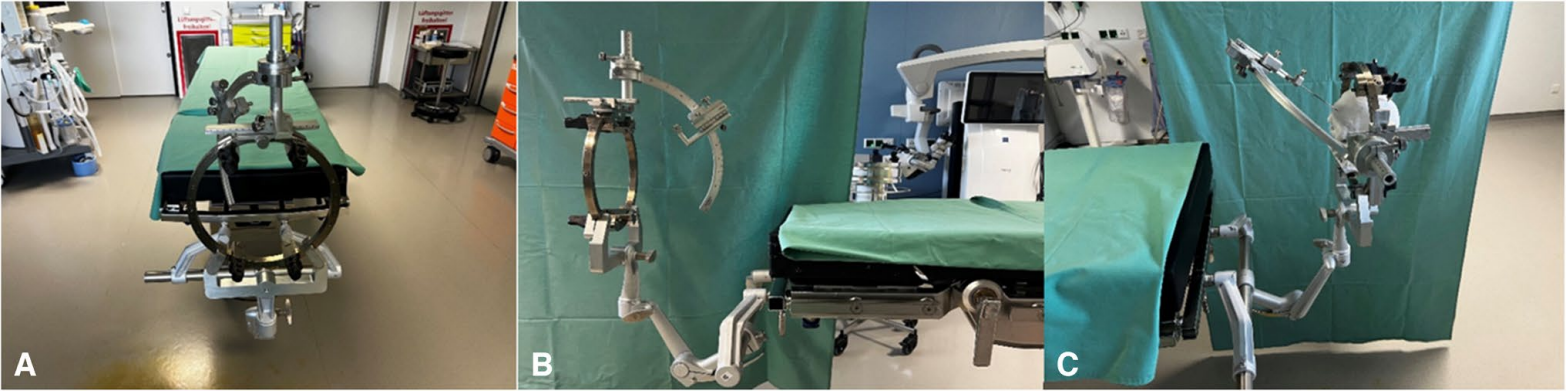
Setting of the using the ZD Inomed frame. **A**: Frame setting in the operation theater, **B**: Fixation of frame, **C**: Frame setting mounted on a model

Stereotactic brain biopsies were performed using either the Leksell Vantage frame (Elekta, Stockhom, Sweden) or the ZD Inomed frame (Inomed Medizintechnik GmbH, Emmendingen, Germany). All procedures were conducted in general anesthesia. For biopsies of the posterior fossa, we adapted the frame setup based on the equipment used: the ZD frame was mounted upside down, inverted and aligned to the orbitomeatal line, or used a frontal approach, while the Leksell Vantage frame was used in a reverse x-axis orientation (Figs. 1 and 2).

Both frame systems were fixed to skull using four screws. Afterwards, a stereotactic CT scan was performed. Planning was conducted using Elements software or before 2017 with iPlan (Brainlab AG, Munich, Germany) based on 3-T MRI scans merged with stereotactic CT for coordinate generation. Patients were positioned in either prone or supine position with an inclined neck in the operating theatre. Due to pre-existing limitations of the phantom, coordinate settings could not be verified in posterior montage. These settings were then transferred to the attached frame system of the patients’ heads.

After sterile preparation, the surgical procedure included a skin incision, skull bure hole, dura coagulation and incision, and corticotomy. Biopsies were performed using a biopsy needle (Inomed Medizintechnik GmbH, Emmendingen, Germany) with a 2.5 mm lateral window, aspirating tissue from four quadrants if no contraindications were present. After needle removal, the trajectory was irrigated with saline and observed for possible bleeding, followed by layered wound closure. All patient received 24-h postoperative Intermediate Care surveillance.

We retrospectively analysed the patient data, demographic aspects, frame settings, anatomical location of lesion, duration of surgical procedure, length of trajectories, histopathological results, pre- and postoperative neurological deficits, postoperative complications and 30-days mortality. Statistical analysis was performed using the Student t-test.

## Results

Between 2006 to 2024, we performed 26 stereotactic biopsies of the posterior fossa in our department. Out of these, 9 were performed using the ZD Inomed frame and the remaining 17 with the Leksell Vantage frame. One patient was excluded from further analysis due to the absence of necessary data.

### Patient data and trajectory objectives

The cohort consisted of 25 patients (14 male, 11 female) with an average age of 60.6 years (range: 36 to 80 years, median: 61 years). Among the lesions, 18 were located in the cerebellum and 7 in the brainstem and cerebellar peduncle. In total, 15 lesions were primarily located on the left side and 10 on the right side of the cerebral midline. The planned and actual trajectorie length ranged from 25.5 to 88 mm. The average trajectory length was 52.2 mm for the ZD Inomed frame and 41.7 mm for the Leksell Vantage system.

While the focus of this analysis in on the technical outcomes rather than a direct comparison of the frame systems, it was observed that the average surgery duration was significantly shorter with the Leksell Vantage frame (32.6 min) compared to the ZD Inomed frame (44.8 min, *p*-value 0.05). The average duration of perioperative procedures was 115 min for the Leksell Vantage frame and 125 min for the ZD-Inomed frame. However, it should be noted that both systems have distinct technical features, and the duration may reflect procedural complexity rather than superiority.

### Histological results

Histopathological results were categorized into three groups: primary brain neoplasms, lymphomas and infections. In our cohort 13 lesions were identified as primary brain neoplasm, 6 as lymphomas, and 4 as infections. Two histopathological samples remained unclear and re-biopsy were not performed due to the sensitive anatomical location and the high likelihood of remaining histologically inconclusive. The demographic and biopsy result data are summarized in Table 1.

**Table 1.**
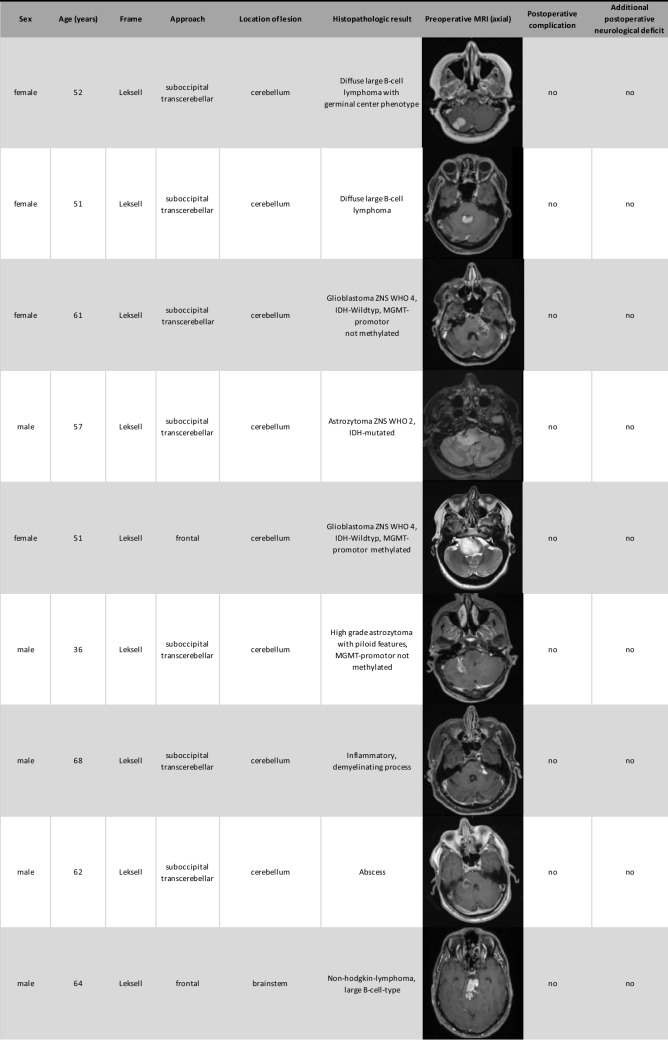

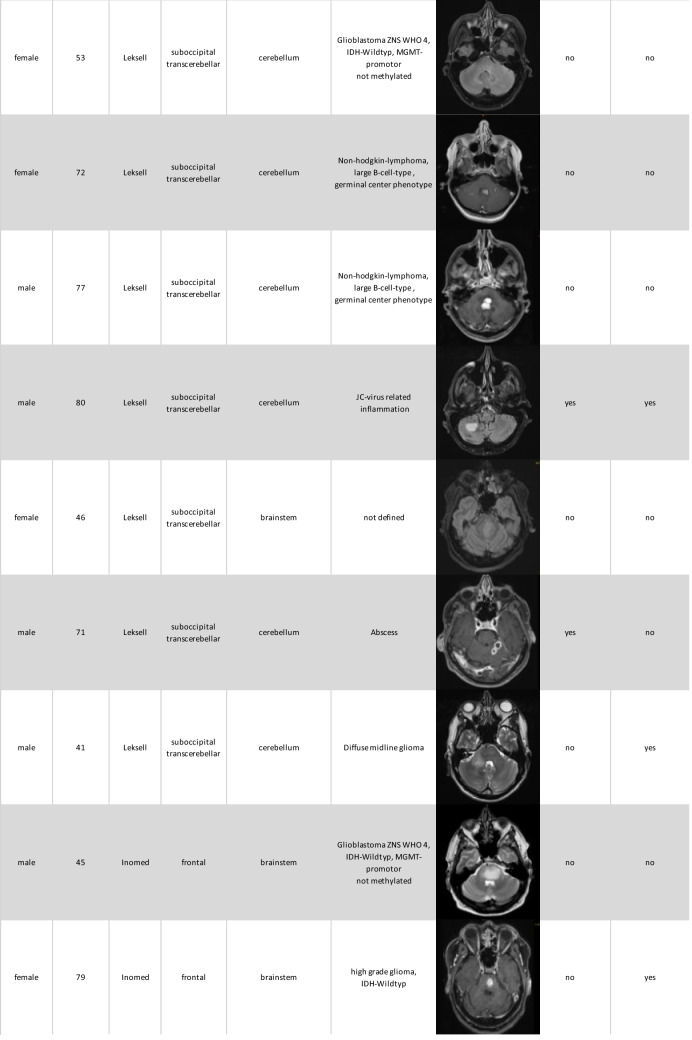

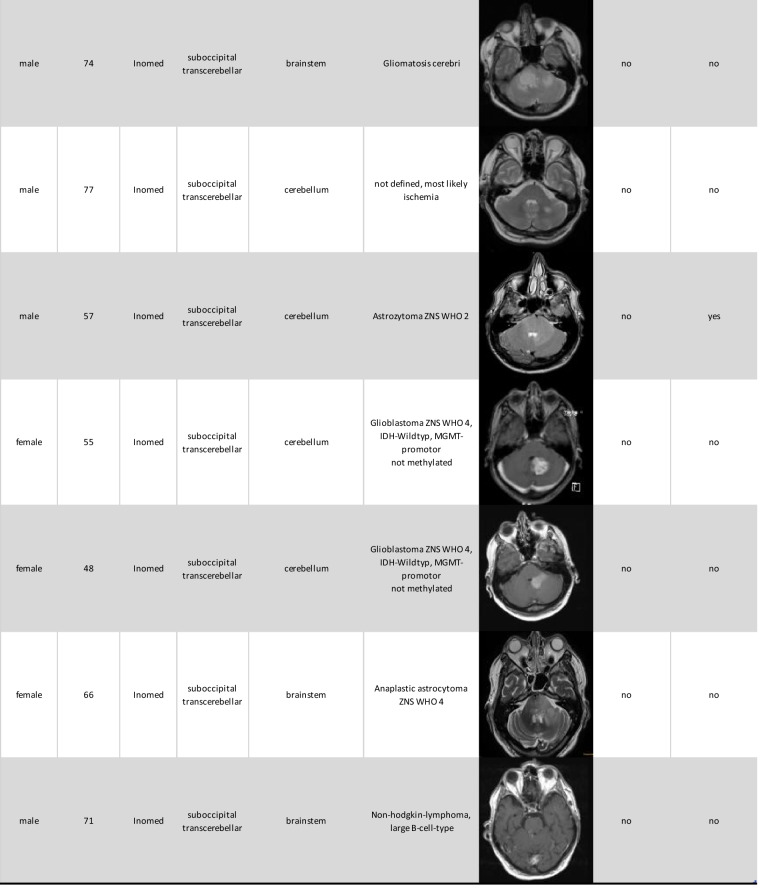
Overview of demographic data, biopsy results, anatomical location and postoperative complications and deficits

### Surgical complications

Postoperative complications included two cases of postoperative bleeding: one managed conservatively (71 years-old male with cerebellar abscess) and the other requiring surgical intervention (80 years-old male with cerebellar JC-virus related inflammation). Additionally, two patients experienced new postoperative focal neurological deficits. One 80 years-old male patient with cerebellar JC-virus related inflammation and a postoperative hemorrhage required surgical intervention and subsequently developed new visual disturbances. The other case involved a 79-year-old female patient with a high-grade glioma of the brainstem who developed dysarthria and paresis of the right upper limb.

In our cohort the overall mortality rate was 34.8%, with a 30-day postoperative mortality rate of 13.0%. There was no significant difference between the two frame groups. In all three cases of death within 30 days post-surgery, the cause was attributed to the progression of the underlying malignancy rather than complications from the biopsy itself.

## Discussion

The transcerebellar approach for stereotactic biopsies is considered a safe and effective method to access cerebellar, pontin and medulla oblongata lesions, as demonstrated by the absence of permanent morbidity and high diagnostic value. However, despite these advantages, stereotactic biopsies of the posterior fossa remain relatively rare. Both our cohort and other published cohorts are limited by small sample sizes [7], which poses challenges for drawing statistically significant conclusions regarding the superiority of different stereotactic frame positions and approaches [9, 8, 11].

### Technical challenges of frame setup

While we observed notable differences in the technical handling of the two systems, we refrain from drawing firm conclusions about their superiority. Specifically, the use of the Leksell Vantage frame necessitates precise prior planning of the trajectory, including the entry point, before mounting the frame system. This requirement, as described by Krüger et al. 2022 arises from the more limited surgical area imposed by the frame system itself compared to the ZD Inomed frame. Furthermore, we observed that the ZD Inomed frame’s head fixation was somewhat unstable during bure hole drilling due to its only posterior one-arm fixation of the frame. To address this, we introduced an additional headrest for support (Fig. 2). Despite the more complex intraoperative setup of the Leksell Vantage frame, the average duration of surgical perioperative procedures was was comparable to, if not shorter than, the duration for the ZD Inomed frame. This may reflect the benefits of the higher precision offered by the Leksell Vantage system.

### Anatomical location and recommended approach

In terms of accuracy, the Leksell Vantage frame allows adjustments to the tenth of a millimeter, compared to the millimeter-level adjustment of the ZD Inomed frame. While this finer adjustment capability may offer potential advantages in specific clinical scenarios, it is important to consider that the typical stereotactic error in frame-based approaches ranges from 1–2 mm (Palys and Holloway, 2018) [12]. Therefore, the practical difference in precision may be less pronounced. Nonetheless, the Leksell Vantage frame remains a valuable tool for targeting sensitive regions like the brainstem, where precision is critical. To ensure the safest approach to the brainstem, thorough evaluation of anatomical fiber tracts is crucial. While a posterior fossa approach might cross fiber tracts with the biopsy needle window, potentially causing significant local trauma, a supratentorial trajectory might be preferable. The latter would align the biopsy needle window to the fiber tracts, reducing trauma but increasing the distance to the target.

Given the complexity and risks involved, it is also worth considering additional technical strategies to further reduce complications, particularly the risk of bleeding. One speculative approach to address intraoperative bleeding risk could be the administration of contrast medium during stereotactic CT in the early arterial phase with a delay time of 35 s and a flow rate of 4 ml/min. This could help visualize early arterial vessels, which would be crucial for trajectory planning to avoid critical vascular structures during the procedure.

Postoperative neurological deficits, such as those observed in our study, are inherently tied to the anatomical location of the lesions, especially in critical areas of the brainstem. To minimize the impact of new neurological deficits and preserve the patient’s quality of life postoperatively, it is crucial to respect anatomical structures and, in particular, the relevant fiber tracts of the brainstem. The integration of advanced imaging techniques, such as tractography, could be a valuable addition to the preoperative planning process. By segmenting critical anatomical structures and visualizing fiber connections, neurosurgeons may be able to refine trajectory planning and potentially reduce the risk of functional impairment.

Current literature includes only one report of using the Leksell Vantage frame for suboccipital transcerebellar biopsy approaches involving 10 patients (Krüger et al. 2022). This study emphasizing that, although the Leksell Vantage frame can be used safely and precisely, meticulous and careful pre-planning is essential. This underscores a significant knowledge gap and highlights the need for further research to determine the most appropriate biopsy approach—whether frontal or suboccipital transcerebellar.

## Conclusion

Our experience with stereotactic biopsies in the posterior fossa underscores the technical complexity and the necessity for highly experienced neurosurgeons to perform these procedures due to the anatatomical vulnerabilities involved. While our study does demonstrate that the Leksell Vantage frame is associated with a shorter surgical duration compared to the ZD Inomed frame, we emphasize that both systems have unique technical advantages that can be tailored to specific patient and lesion characteristics. Rather than focusing on a direct comparison of the systems, it is crucial to approach each case with individualized planning based on the lesion’s location and the surrounding anatomical structures.

The choice of surgical approach—whether frontal or transcerebellar—should be made with careful consideration of the lesion’s location and potential impact on surrounding anatomical structures. At our institution, we typically employ a frontal biopsy approach for brainstem lesions located higher than the middle cerebellar peduncle to minimize trauma to the longitudinal fibers. Conversely, for cerebellar processes or lesions situated below the middle cerebellar peduncle, we favor a transcerebellar route. We also recognize the potential for improving surgical outcomes by incorporating advanced imaging techniques such as tractography and contrast-enhanced stereotactic CT, which could further refine trajectory planning and reduce complication rates.

This Technical Note aims to provide detailed procedural insights and practical guidance for neurosurgeons. It is our hope that sharing our experiences will contribute to the refinement of stereotactic biopsy techniques in the posterior fossa and inspire further research to enhance the safety and efficacy of these challenging procedures.

## Data Availability

No datasets were generated or analysed during the current study.
